# Analyzing whole genome bisulfite sequencing data from highly divergent genotypes

**DOI:** 10.1093/nar/gkz674

**Published:** 2019-08-08

**Authors:** Phillip Wulfridge, Ben Langmead, Andrew P Feinberg, Kasper D Hansen

**Affiliations:** 1 Center for Epigenetics, Johns Hopkins School of Medicine, 855 N. Wolfe St, Baltimore, MD 21205, USA; 2 Department of Computer Science, Johns Hopkins University, 3400 N. Charles St, Baltimore, MD 21218, USA; 3 Department of Medicine, Johns Hopkins School of Medicine, 855 N. Wolfe St, Baltimore, MD 21205, USA; 4 Department of Biomedical Engineering, Whiting School of Engineering, 3400 N. Charles St, Baltimore, MD 21218, USA; 5 Department of Mental Health, Johns Hopkins Bloomberg School of Public Health, 624 N. Broadway, MD 21205, USA; 6 Department of Biostatistics, Johns Hopkins Bloomberg School of Public Health, 615 N. Wolfe St, Baltimore, MD 21205, USA; 7 McKusick-Nathans Institute of Genetic Medicine, Johns Hopkins School of Medicine, 733 N. Broadway, Baltimore, MD 21205, USA

## Abstract

In the study of DNA methylation, genetic variation between species, strains or individuals can result in CpG sites that are exclusive to a subset of samples, and insertions and deletions can rearrange the spatial distribution of CpGs. How to account for this variation in an analysis of the interplay between sequence variation and DNA methylation is not well understood, especially when the number of CpG differences between samples is large. Here, we use whole-genome bisulfite sequencing data on two highly divergent mouse strains to study this problem. We show that alignment to personal genomes is necessary for valid methylation quantification. We introduce a method for including strain-specific CpGs in differential analysis, and show that this increases power. We apply our method to a human normal-cancer dataset, and show this improves accuracy and power, illustrating the broad applicability of our approach. Our method uses smoothing to impute methylation levels at strain-specific sites, thereby allowing strain-specific CpGs to contribute to the analysis, while accounting for differences in the spatial occurrences of CpGs. Our results have implications for joint analysis of genetic variation and DNA methylation using bisulfite-converted DNA, and unlocks the use of personal genomes for addressing this question.

## INTRODUCTION

DNA methylation is a key epigenetic mark that has become widely implicated in human development and disease (1,2). Accurate determination of methylation at CpG dinucleotide positions across the genome is critical for understanding its association with functional regulation. Multiple techniques currently exist to perform this measurement, each with varying degrees of genomic coverage and depth. One gold-standard method is whole-genome bisulfite sequencing (WGBS), which pairs bisulfite conversion of cytosine residues with next-generation sequencing (3). To quantify methylation in WGBS, bisulfite converted reads are compared to an in silico bisulfite-converted genome sequence, referred to as the reference genome (3–5). At each CpG site in the reference genome, an aligned read is called as unmethylated if the sequence is TG (indicating bisulfite conversion) and methylated if the sequence is CG (indicating protection by the methyl group). Statistical packages such as BSmooth (4) can then integrate this data across larger regions to estimate and compare overall methylation patterns between sample groups.

It is well understood that a CG-to-TG mutation is the most common dinucleotide mutation in the mammalian genome, due to the high rate of spontaneous deamination at methylated CpGs (6–8). This effect becomes particularly pronounced in animal models such as mice: inbred strains can be separated by up to millions of nucleotide variants and indels, a large proportion of which affect CpG dinucleotides. This presents a substantial pitfall when analyzing bisulfite converted DNA: if the sample genome has a CG-to-TG (or CG-to-CA) variant relative to the reference genome sequence, reads aligning to the variant using standard alignment approaches will produce unmethylated calls without inducing any alignment mismatches (9). This will result in an excess of 0% methylated ‘sites’ at locations where there is actually no CpG sequence.

The problematic effect of C/T variants on methylation quantification is widely recognized, and various methods exist to compensate. While it is possible to identify variants in bisulfite converted data by considering reads aligned to the opposite strand (9,10), it is self-evident that the best solution is to simply align each sample to its own genome sequence, assuming such sequence is available. Variants of this strategy are indeed commonly used in analysis, i.e. with alignment to approximate personal genomes; this has been done both in plants (11–13) and mammals (14,15). However, while both the problem and the optimal solution are recognized, the magnitude of bias is not well understood or described. Filling this gap in understanding is especially important for experiments involving a large number of distant or mixed genotypes. In these experiments, knowing whether CpG variation presents a substantial enough bias to necessitate generating tens to hundreds of personal genomes, which can demand substantial computational resources, is of high importance.

But the effect of C/T variants goes beyond alignment. Specifically, if each sample is aligned to a separate genome, a critical issue arises on how comparisons should be made across different genomes, whose coordinates will be separated by insertions and deletions, as well as how strain- or sample-specific CpGs (that is, CpGs only existent in a subset of individuals) should be handled in analysis. Common methodologies treat sample-specific CpGs as incomparable and either drop CpGs not covered in all samples, or drop samples for which a CpG does not exist. However, in the face of mounting evidence that sites of hypervariable CpG mutation are strongly related to species-specific disease and phenotype (16), failing to account for the implications of CpG loss on DNA methylation may instead represent a substantial loss of information. As a recent example, a study in humans (14) produced approximate sample-specific genomes by substituting single nucleotide variants into a common reference genome, then analyzed methylation differences at individual CpGs; for each of these CpGs, samples in which the CpG was lost were discarded from analysis. In an analysis that drops sample-specific CpGs, however, a genomic location where half the samples had a CpG with high methylation, while the other half had lost the CpG, would not be identified as a position of variable methylation, a questionable conclusion.

Here, we address two basic questions. First, how essential is it to use personalized genomes, since acquiring such data comes at a cost? Second, how should different coordinate systems across reference genomes be reconciled, and how can strain- or sample-specific CpGs be factored into comparisons? We examine sequence variation and methylation data from two inbred but highly divergent mouse strains: C57BL/6J, upon which the mouse reference genome is based, and the wild-derived CAST/EiJ. We describe patterns in strain-specific CpG variation, and quantify the substantial effect of ignoring this variation when performing alignment and methylation quantification. Next, we propose a smoothing-based method that allows strain-unique CpGs to contribute to identification of differentially methylated regions (DMRs), and show that doing so increases power. At last, we apply our method to a human disease context using publicly available cancer data.

## MATERIALS AND METHODS

### Short-read alignment

Alignment and CpG read-level measurement were performed using Bismark version 0.16.1 (5) and Bowtie2 version 2.1.0 (17). Reference genomes for BL6 and CAST were generated from their corresponding FASTA files (build 37), obtained from UNC Systems Genetics. Sequencing reads were first trimmed using Trim Galore! version 0.3.7 using default options, then aligned with Bismark using options –bowtie2 –bam. BAM output files were merged and sorted in preparation for methylation extraction using Samtools version 1.3 (18). Read-level measurements were obtained using the Bismark methylation extractor, with options -p –ignore 5 –ignore_r2 5 –ignore_3prime 1 –ignore_3prime_r2 1. Read measurements were modmapped if applicable (see section below), then converted to BSseq objects in R version 3.3.0 using the bsseq package (4). When creating BSseq objects, forward- and reverse-strand reads were combined for each CpG. Deduplication rates were measured using Bismark deduplicate_bismark. Bisulfite conversion efficiency was calculated for each sample by subtracting the mean methylation across the spike-in lambda sequence from 100%.

As alternatives to Bismark, we also performed alignments with bwa-meth version 0.2.2, which uses BWA version 0.7.12 (19) as its aligner, and BSMAP version 2.90 (20), using the same reference FASTA files and trimmed sequencing reads. Read-level measurements were quantified using MethylDackel version 0.3.0-3-g084d926, which is an equivalent to Bismark’s methylation extractor for the BWA and BSMAP pipelines.

### Coordinate mapping between strains

To facilitate direct comparison of CpGs between strains, we converted genomic coordinates using the ‘modmap’ package from UNC Systems Genetics (21). This package functions similarly to the liftOver tool (22) on the UCSC Genome Browser, and was used to convert CpG locations in the CAST genome to their corresponding coordinates in the BL6 genome, and *vice versa*. CpGs containing negative positions on either strand in the modmap output (indicating the location resided within an insertion or deletion in the other strain) were discarded.

### Focal methylation analysis

We used the BSmooth pipeline as implemented in the bsseq package from Bioconductor (4), as employed previously (23,24). For small DMR analysis, the data was smoothed using BSmooth with default parameters (ns = 70, *h* = 1000, maxGap = 10^8^). For large-scale block analysis, data were smoothed with parameters (ns = 500, *h* = 20 000, maxGap = 10^8^). Following smoothing, we used t-statistics (cutoff = 4.6, maxGap = 300) to obtain putative DMRs and (cutoff = 2, maxGap = 10 000) to obtain putative blocks, only analyzing CpGs where at least three samples in each group had a coverage of at least 2. Significance was assessed using a stringent permutation approach as described previously (24). Specifically, we used permutations which balanced the two strains (i.e. each permutation has two BL6 and 2 CAST samples in each group); there are 18 such permutations. For each DMR we calculated how many permutations contained a better null DMR; dividing by the total number of permutations gives us the quantity we call gFWER. To be precise we say one DMR is ‘better’ than another if it has a greater number of CpGs as well as a greater total sum of t-statistics across all CpGs in the DMR. By comparing each putative DMR to all null DMRs in all permutations we control for multiple testing and familywise error rate. The interpretation of a gWER of 1/18 for a given DMR is that in 1 out of 18 permutations do we see a better null DMR *anywhere* in the genome.

### Analysis of human cancer-normal methylation

Sequencing reads for human brain and U87MG cell line samples were obtained from GEO accession GSE52272 (25). Variant information on the U87MG cell line was obtained from GEO accession GSE19986 (26). As indicated by a 2018 correction, this variant data applies to a distinct U87 cell line obtained from ATCC; this was the same cell line used by the methylation study.

Using the U87MG variant data and the hg19 genome FASTA, we generated a pseudogenome for U87MG via the modmap tool suite. Sequencing reads were trimmed and aligned as described in short-read alignment; the normal sample was aligned to hg19, and the U87MG sample was aligned separately to hg19 and to the U87MG pseudogenome. Samples were placed into the hg19 coordinate system via modmap, heterozygous CpG sites removed and smoothed over all remaining CpGs, including remaining genotype-specific CpGs, with parameters (ns = 70, *h* = 1000, maxGap = 10^8^).

## RESULTS

### WGBS data on mouse strains

As an extreme example of massive CpG variation between genomes, we examined sequence and methylation data from two inbred mouse strains. C57BL/6J (BL6) is the standard laboratory strain and the basis of the mm9 reference genome, whereas CAST/EiJ (CAST) is wild-derived and highly divergent, as evidenced by a large number of single nucleotide variants (20 539 633) and indels (8 171 218) in its reference sequence (Supplementary Methods). Methylation data were generated via WGBS on liver samples from each strain (*n* = 4 per strain); the initial purpose of this experiment was to identify DMRs between strains. We generated sequencing data at relatively low coverage and high bisulfite conversion, and aligned all samples to both genomes using Bismark (‘Materials and Methods’ section, Supplementary Methods, Tables 1 and 2).

**Table 1.** Number of reads and alignment statistics

Sample	nReads	aRate^a^	CpG^c^	aRate^a^	CpG^b^
BL6_1	165 455 305	55.7	5.8	49.2	4.7
BL6_2	155 446 345	55.1	5.5	48.6	4.5
BL6_3	165 687 191	60.3	6.8	52.9	5.5
BL6_4	172 926 402	58.9	6.7	52.0	5.5
CAST_1	171 357 014	62.8	6.3	71.0	6.4
CAST_2	161 768 892	55.8	6.1	63.1	6.3
CAST_3	154 973 730	56.0	5.5	63.4	5.7
CAST_4	188 134 260	48.1	6.0	54.5	6.2

^a^alignment rate, ^b^coverage of CpGs.

**Table 2.** Sequencing quality measures

Sample	Conv %^a^	Dup %^b^ (BL6)	Dup %^b^ (CAST)
BL6_1	99.7	9.41	9.10
BL6_2	99.7	7.44	7.16
BL6_3	99.7	15.26	14.49
BL6_4	99.8	9,80	9.40
CAST_1	99.7	5.26	5.44
CAST_2	99.7	9.17	9.61
CAST_3	99.7	7.82	8.11
CAST_4	99.7	7.82	8.09

^a^conversion efficiency using lambda spike-in, ^b^duplication rate of aligned reads.

### CpG variation across the mouse genome

**Figure 1.**
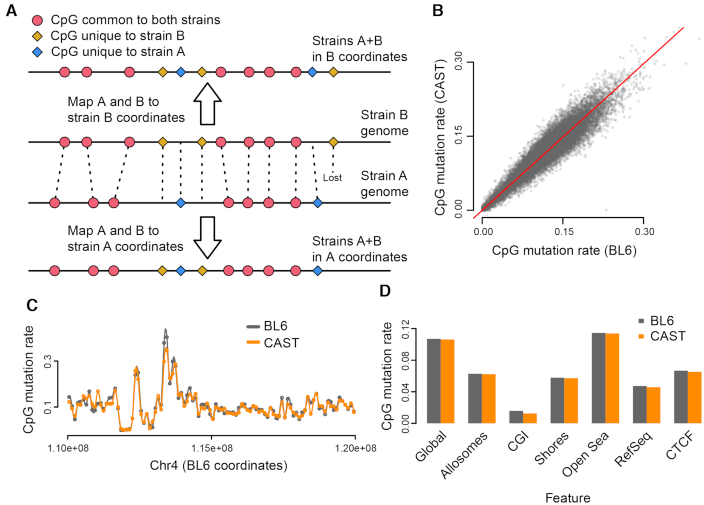
Characteristics of strain-specific CpG mutations. (**A**) A representation of whole-genome alignment between two strains, A and B. The CpGs present in the two strains can be represented in both coordinate systems; CpGs are either shared between strains (red), mutated (yellow or blue) or ‘lost’ due to indels that render the coordinate unmappable (rightmost yellow). (**B** and **C**) The CpG mutation rate (proportion of strain-specific CpGs relative to total CpGs) calculated in 100 kb bins, is comparable between BL6 and CAST strains, both genome-wide (B) and locally (C). (**D**) The CpG mutation rate across different genomic features.

We first examined differences between the DNA sequences of BL6 and CAST in order to quantify and characterize CpG variation. To facilitate accurate sequence comparisons between these two genomes, which do not share coordinate systems, we used the modmap tool (21). This tool functions similarly to UCSC’s liftOver (22) to convert a set of genomic locations to their corresponding coordinates in another strain. Using this tool, we can determine whether a CpG in one strain retains its sequence in the other strain, contains a mutation or cannot be accurately mapped, which occurs when an indel over a CpG results in an ambiguous position after modmap conversion (Figure 1A).

Using modmap and the FASTA files for mm9 (BL6) and CAST, we extracted and tabulated the forward-strand dinucleotide sequences corresponding to the 21.3 million CG dinucleotides in BL6 (Table 3). Approximately 19 million CGs from BL6 are shared by CAST; 1.64 million are mutated to either TG or CA, with another 0.54 million mutated to GG/CC and AG/CT. Another 100k CpGs could not be accurately mapped from BL6 to CAST due to strain indels over the sequence. We observed similar results when we performed the reverse analysis: of 21.4 million CGs in CAST, about 2.3 million are unique to CAST, with a differing or unmappable sequence in BL6 (Table 3).

**Table 3.** Number of CpGs in different strains

	Autosomes		Allosomes	
	BL6	CAST	BL6	CAST
Common CG	18 140 628		916 392	
TG/CA in other strain	1 601 446	1 669 901	43 330	45 041
Lost in other strain	522 845	535 219	15 394	15 838
Unmappable	99 781	90 356	2676	2438
Total	20 364 700	20 436 104	977 792	979 709

Based on our observations, we calculated the ratio of strain-unique to total CpGs, or CpG ‘mutation rate’, at roughly 2.3M/21.3M=10.7%, compared to the overall genomic mutation rate of 1.2% (Methods). This high rate of CpG mutation is consistent with the tendency of methylated C positions to undergo spontaneous deamination to T (6–8). The CpG mutation rate is not uniform throughout the genome, but can fluctuate across local regions. Interestingly, however, the proportion of BL6-unique CpGs in any given region tends to closely match that of CAST-unique CpGs (Figure 1B and C). The CpG mutation rate also varies between different functional regions, for example being much lower in CpG islands and somewhat lower in promoter and CTCF sites (Figure 1D).

### Not using strain specific genomes induces dramatic bias

**Figure 2.**
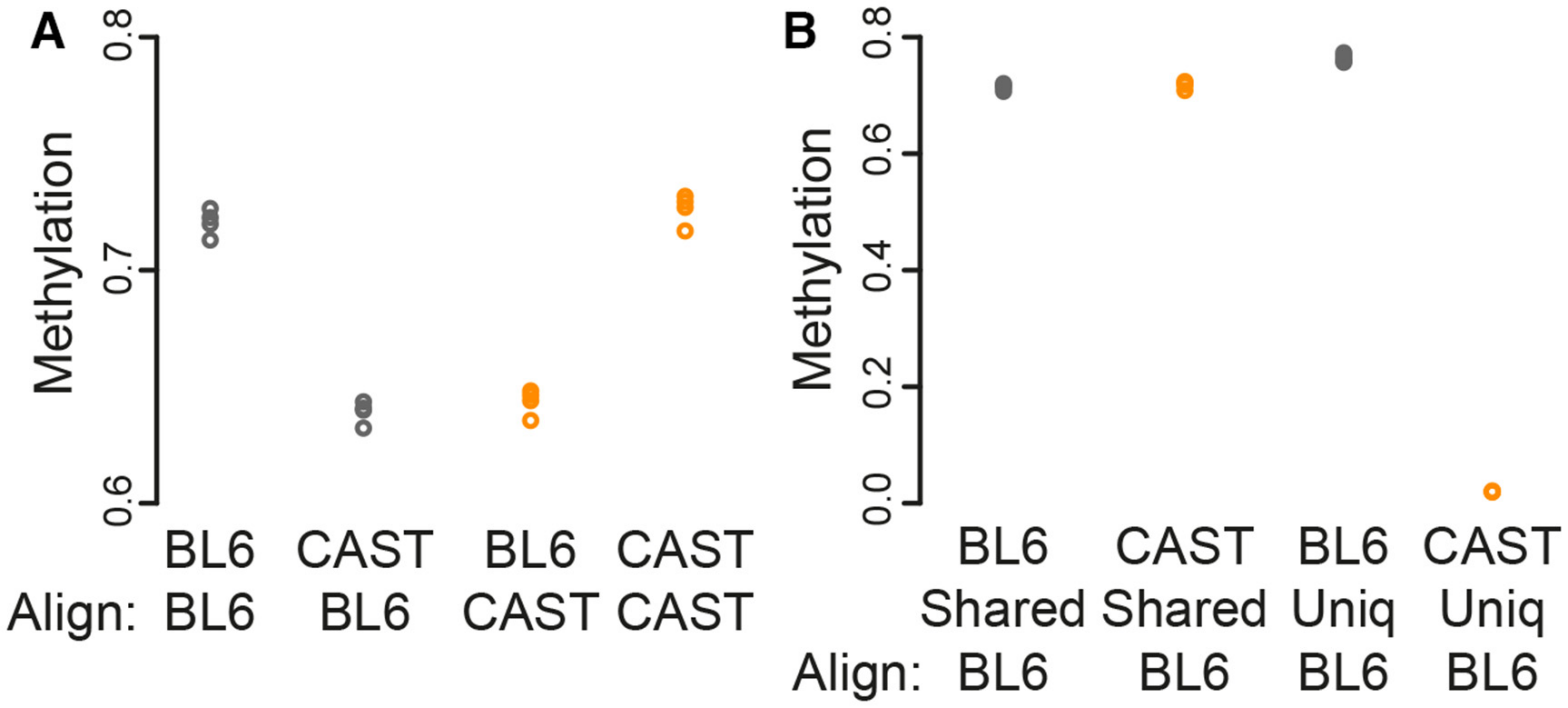
Effect of CpG variation on global methylation. (**A**) Global methylation estimated for four samples each of two different mouse strains (BL6 and CAST) aligned to either of the two reference genomes. Samples aligned to the strain-specific genome display higher global methylation compared to the same sample aligned to a distant reference genome. (**B**) Average methylation for different subsets of CpGs within BL6 and CAST samples, all aligned to the BL6 reference genome. In CAST samples, CpGs unique to BL6 are quantified as having 0% methylation.

To show the impact of not using personalized genomes for alignment, we first aligned sequencing reads from both strains to the standard BL6/mm9 reference genome, which represents an analysis pipeline making no adjustment for genotype differences. When we computed the average methylation across all read-covered autosomal CpGs in the reference, which we call global methylation, the two strains showed a large difference, with the CAST strain’s estimates lower by over 7.6% (Figure 2A, *P* < 1.3 × 10^−6^). This difference is comparable in magnitude to levels previously observed between tumor and normal colon (23) and associated with EBV-mediated oncogenesis (24), and is far larger than what we could realistically expect from a comparison between strains or individuals. Note that global methylation is an average across millions of CpGs, and thus unlikely to be affected to this extent by local differences in coverage. This observation was reversed when samples were aligned to the CAST genome (Figure 2A), confirming this was caused purely by the choice of reference genome used for alignment. We term this bias ‘quantification bias’; in this case, quantification bias introduced by using the wrong genome can be estimated at 7–9%.

As expected, the observed discrepancy in global methylation can be explained by CpG sequence variation. When we categorized CpGs by whether they were present in both strains, we observed a large methylation difference only in the CpGs unique to BL6, with the computed methylation levels of CAST samples in those CpGs being virtually 0% (Figure 2B). This is consistent with the observation that most BL6-unique CpGs have a TG/CA sequence in CAST (Table 3), and shows that the bias in global methylation is predominantly caused by assigning a methylation percentage of 0% to these ‘lost’ CpGs. Unsurprisingly, this bias is addressed by alignment to personalized reference genomes: CAST samples aligned to the CAST reference have a global methylation roughly equivalent to that of BL6 samples aligned to mm9 (Figure 2A).

Because the bias introduced by using an incorrect reference genome derives from the methylation estimation step of an alignment pipeline, rather than some property of the sequencing reads themselves, it occurs regardless of the alignment method used. We confirmed this by performing alignment of the same sequencing reads from all samples, to both BL6 and CAST reference genomes, via two alternative aligners, BWA (19) and BSMAP (20). In both cases, global methylation remained highly skewed, with samples aligned to a distant reference consistently showing 8–9% lower methylation (Supplementary Figure S1).

**Figure 3.**
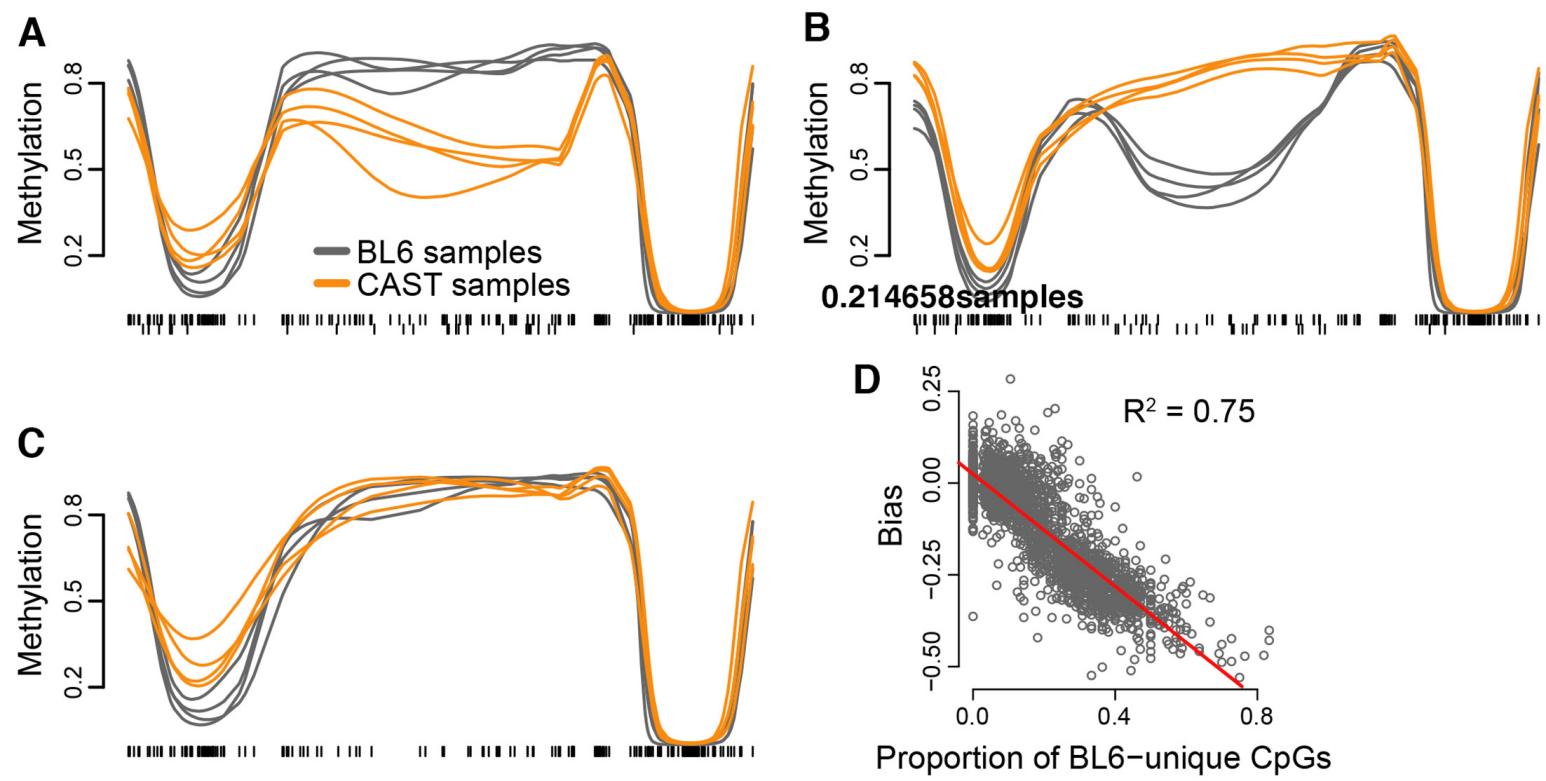
Quantification bias causes false-positive focal methylation changes. The example region pictured in (**A**) is identified as a DMR in mm9-aligned analysis, due to multiple BL6-unique CpGs (downwards ticks) that are read as having near-0% methylation in CAST samples. The DMR is reversed in direction if all samples are aligned to CAST due to the same effect occurring with CAST-unique CpGs (**B**), and no differential methylation is observable once these sites are removed from analysis (**C**). The magnitude of bias in such false-positive DMRs (i.e. the change in apparent differential methylation when unique CpGs are removed) is highly correlated with the regional level of CpG variation (C).

As with global methylation, analysis of focal changes was also strongly impacted by quantification bias. We used the BSmooth pipeline (4) to identify small DMRs between BL6 and CAST; these range from hundreds to a few thousand bases in width. Performing this analysis on mm9-aligned samples, without adjustment for CpG variation, identified 2865 DMRs that passed our cutoff criteria, which includes a measure of family-wise error rate (‘Materials and Methods’ section, Supplementary Table S1, Figure 3A). However, the vast majority of these DMRs were artifacts of misleading 0% methylation measurements in CAST samples: when we simply removed BL6-unique CpGs prior to smoothing as a rudimentary form of adjustment, many observed mean differences in DMRs were diminished or absent entirely—only 554 (19.3%) were still identified as significant by the new analysis (Figure 3C).

As further evidence of the impact of bias, many DMRs reversed in direction when we aligned all samples to the CAST genome, as BL6 samples now had a disproportionate number of 0% methylation measurements (Figure 3B). In all of these cases, the magnitude of a given DMR’s methylation discrepancy was highly correlated with the number of unique CpGs in the region (Figure 3D). This analysis shows that quantification bias can cause false positives that vastly outnumber the potential true positive strain differences. It also suggests the presence of a modest number of genomic regions which are truly differentially methylated between the two strains—the original purpose of generating the data.

We have previously described an analysis method for identifying large-scale changes in DNA methylation, at the scale of 100 kb or more (23). Applying this method to data aligned to BL6 identified 2354 regions comprising 347 Mb and covering 1.9M CpGs. For data aligned to CAST, we found 4199 regions comprising 1101 Mb and 6.4M CpGs. Again, the direction of change reversed entirely with choice of reference genome: 98.6% of regions were hypermethylated in BL6 when aligned to BL6, whereas 99.3% were hypermethylated in CAST when aligned to CAST (Supplementary Figure S2). This is unsurprising given that such large regions contain a large number of strain-unique CpGs, mirroring the consistent bias of global methylation calculations. Unlike the result for small scale changes, this analysis suggests that there are no true large-scale changes in DNA methylation between BL6 and CAST.

Based on these observations, we conclude that the use of an incorrect reference genome introduces clear and substantial quantification bias into DMR finding strategies. Therefore, usage of personalized reference genomes, or at least careful consideration of adjustments for CpG variation, is imperative when performing WGBS analysis on samples with distant genotypes.

### Regional smoothing over strain-unique CpGs increases power for focal analysis

We now discuss potential strategies for dealing with sites of CpG variation. Essentially, two options exist: remove all CpG sites only present in one genotype, or somehow include those sites indirectly. The former strategy is straightforward to implement and widely used (27), but (as we have noted above) sacrifices a substantial amount of information. In contrast, a method that could incorporate strain-unique CpG data into regional finding would increase analytical power.

Fortunately, existing methods for regional analysis are already primed to handle data from genotype-unique CpGs. For example, BSmooth (4) smooths methylation data over all covered CpGs by default, without a need to manually designate genotype-unique CpGs: CpG sites that are nonexistent in a certain sample are simply treated as zero-coverage, and thus are excluded from smoothing in that sample. The BSmooth process yields a set of continuous methylation curves across the whole genome, which can then be evaluated within just a set of common CpGs; however, data from nearby genotype-unique CpGs influence the imputed methylation values at these common CpGs, and in this way contribute to the final analysis.

Therefore, we propose a smoothing-based algorithm that integrates strain-unique CpGs as an improvement to existing methods. Specifically, what we will refer to as the *unique-removed* and *unique-included* pipelines can be outlined as follows:

- Map reads to personalized genomes and quantify methylation.
- Using whole-genome alignment tools, such as modmap or liftOver, place CpGs from each sample into a common coordinate space.
- Filter out any CpGs that are not shared across all samples (*unique-removed*); or, retain all strain-unique CpGs (*unique-included*).
- Smooth methylation values to obtain personalized, continuous methylation profiles for analysis.

To test and compare our method, we applied the unique-removed and unique-included analysis pipelines to the same WGBS data of BL6 and CAST mice from previous sections. We aligned all samples to their own reference genome, quantified methylation, and used modmap to place all samples in the mm9 coordinate system. For the unique-removed analysis, we removed the 4.4 million CpGs unique to either BL6 or CAST, then smoothed over the remaining 19 million CpGs. In contrast, all 23.4 million CpGs were retained and smoothed in the unique-included analysis, as per the algorithm outlined above.

As before, focal analysis was performed to identify small DMRs between strains. The unique-included analysis found 976 DMRs passing our cutoff criteria (Methods, Supplementary Table S2). DMRs were strongly enriched for overlaps with various histone marks, transcription factors and other genomic features of interest obtained from ENCODE, suggesting that these represent true regions of interest for investigating strain differences between BL6 and CAST (Supplementary Tables S3 and 4). We selected two top DMRs for pyrosequencing validation (around the *Aldh16a1* and *Tdgf1* genes), confirming large and significant strain differences in both regions (Supplementary Figures S3 and 4, additional comments in Supplementary Discussion, Supplementary Methods andSupplementary Table S5).

We next observed that less differential methylation was identified in the unique-removed analysis, which found only 716 DMRs (Supplementary Table S6). This supported our assertion that integrating all available CpG data would result in a higher identification rate. Interestingly, however, there was no complete overlap between analyses. 655 DMRs were shared between unique-included and unique-removed DMRs, as defined by simple regional overlap (at least one CpG in common); 321 DMRs in the unique-included analysis were not present in the unique-removed analysis, and 61 unique-removed DMRs were similarly not identified by unique-included analysis.

**Figure 4.**
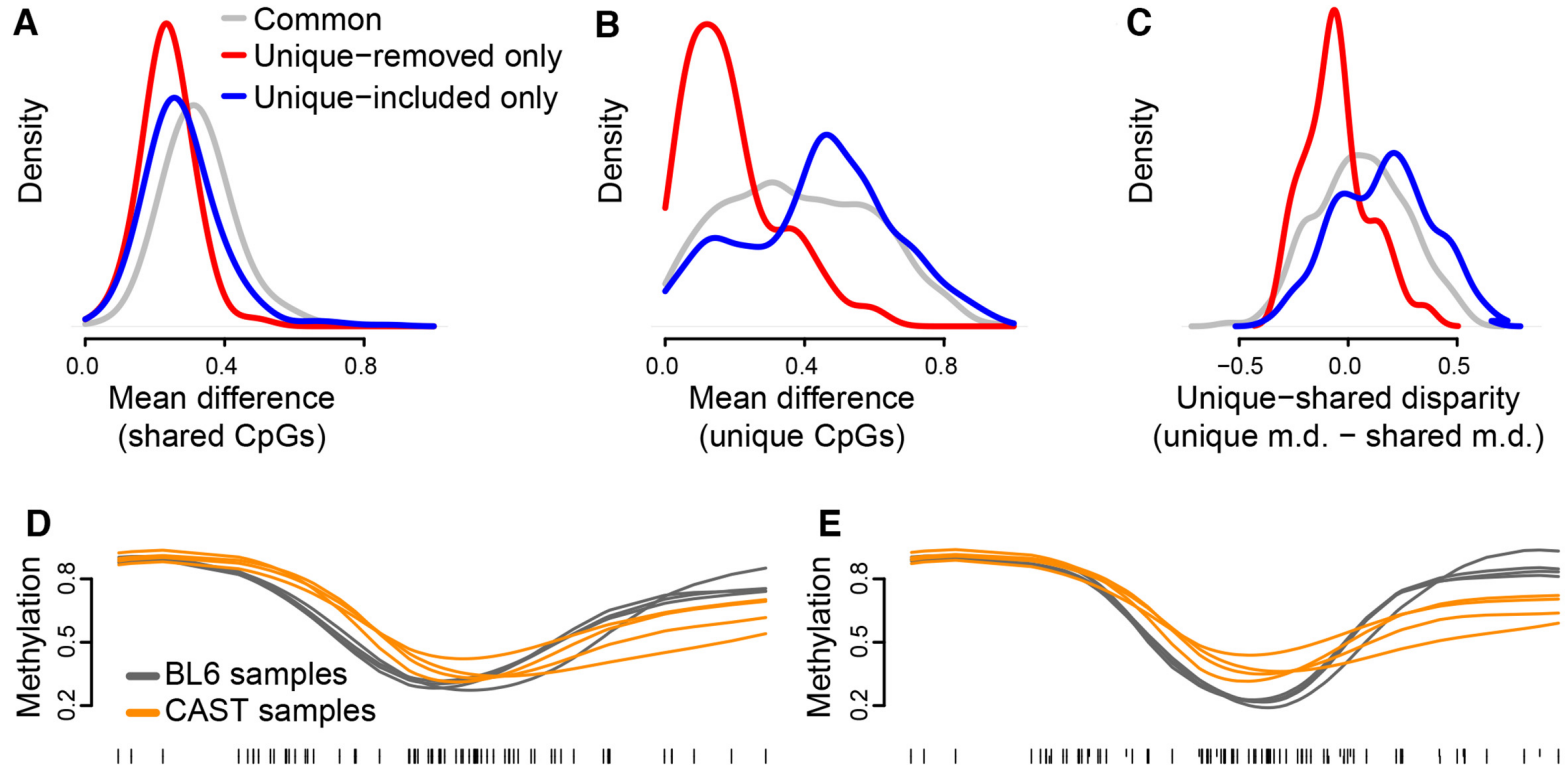
Including strain-unique CpGs identifies additional DMRs in focal analysis. (**A**) A density of the mean methylation differences across three sets of DMRs. For each DMR, the mean methylation difference was computed using unsmoothed methylation values for CpGs present in both strains. (**B**) Like (A) but the mean difference is computed only using CpGs unique to either strains. (**C**) A density of the difference between the mean methylation differences in (B) and (A). (**D** and **E**) An example DMR chosen from those only identified in the unique-included analysis shows no observable difference between strains in the unique-removed analysis (D) but a larger difference in the unique-included analysis (E).

As an example of a DMR only identified by unique-included analysis, we plotted a region within the *Eif2ak3* gene (Supplementary Figure S5), whose variants have been associated with Wolcott-Rallison syndrome and diabetes (28). In the unique-removed analysis, smoothing imputes only a 7% methylation difference between BL6 and CAST, which is below the 10% detection threshold for DMR finding (Figure 4D); in contrast, the unique-included analysis annotates a 14% difference (Figure 4E). Notably, this region contains three strain-unique CpGs, two in BL6 and one in CAST, between which there is a 47% difference in raw methylation levels, and which thus contribute strongly to the calculated strain difference across the region. Pyrosequencing validation across this region confirmed significant methylation strain differences, including between two of the strain-unique CpGs (Supplementary Figure S5).

By definition, the difference in DMR finding between analyses must be exclusively attributable to the presence or absence of strain-unique CpG contributions to regional methylation. One potential mechanism by which this might occur would be strain-unique CpGs exhibiting larger differences between strains compared to nearby shared CpGs, as is suggested to occur by our example region above (Figure 4D and E). These differences would contribute to imputation across the region only if included; thus, the region would register a methylation difference passing the cutoff in the unique-included analysis, but not in the unique-removed analysis. Conversely, strain-unique CpGs with comparable methylation levels, across a region with otherwise large differences in shared CpGs, might drive a reduction in the overall strain difference of the region. However, this reduction would be missed by a unique-removed analysis.

Using this reasoning, we took the full list of DMRs identified across both analyses; then for each genomic region, we obtained raw methylation values across that region from the unique-included BSmooth object, which contains all CpGs. This allowed us to characterize and compare methylation differences in shared versus strain-unique CpGs for all DMRs, including those from the unique-removed analysis. Mean differences across shared CpGs were comparable in DMRs identified in only one analysis, and somewhat higher in DMRs identified in both analyses (Figure 4A); this could indicate that DMRs identified by both analyses had high enough methylation differences in shared CpGs alone to consistently pass our cutoff. In strain-unique CpGs, however, DMRs identified only in the unique-included analysis had a relatively higher proportion of large strain methylation differences; in contrast, those DMRs identified only in the unique-removed analysis had consistently low strain-unique differences, and DMRs identified in both analyses were evenly distributed (Figure 4B).

As a final way of measuring the contribution of strain-unique CpGs to DMRs, we calculated a value that we termed ‘unique-shared disparity’, defined as (mean difference of unique CpGs)—(mean difference of shared CpGs). That is, a unique-shared disparity >0 would indicate that the mean difference in strain-unique CpGs was greater across a region than the mean difference in shared CpGs, and *vice versa*. Our visualization of unique-shared disparity solidified our prediction: most unique-included-only DMRs had disparity >0, while the majority of unique-removed-only DMRs had a disparity <0 (Figure 4C). From this result, we conclude that our unique-included algorithm increases power: a sizable number of regions in the genome had differential methylation driven mainly by strain-unique CpGs, and thus could only be identified via our method. Conversely, strain-unique CpGs could also mitigate methylation differences across a region; a unique-removed analysis would fail to account for this and calculate an inflated mean difference, resulting in false-positive identifications.

### Strategies for methylation analysis in the absence of personal genomes

In the previous sections, we have discussed methylation analysis of divergent genomes under the assumption that accurate reference genomes exist for each unique genotype. Though this is the case for most inbred mouse strains, which are extensively curated, we can envision situations (e.g. studies in other species) where such personal references are unavailable; in these cases, other measures must be taken to mitigate quantification bias.

One possibility for addressing CpG variation is genotyping of samples using bisulfite converted data, for which various methods exist (9,10,29). However, these methods have been evaluated on human data, where the number of variants in an individual is roughly an order of magnitude smaller than that between the more distant mouse strains—3.3 million SNPs in human (30), compared to 20.5 million SNPs between BL6 and CAST (‘Materials and Methods’ section). Furthermore, these methods require high coverage to work. Nevertheless, we proceeded to assess this approach for our data.

To test the feasibility of bisulfite genotyping in our data, we pooled our four genetically identical CAST replicates into one meta-sample with 24× coverage, aligned to the BL6 genome and genotyped using BS-SNPer (10); we then examined how much of CAST’s CpG variation was accurately identified. From our CAST alignment data, we know that CAST-unique CpGs in the meta-sample are well-covered by reads, with 2.17M of 2.26M covered at the recommended 10× or higher; despite this, however, BS-SNPer performed poorly at identifying these CpGs, only calling 1.15M (51%) correctly (Table 4). Identification of BL6-unique CpGs, which are lost in the CAST metasample, was even less efficient, with only 542K of 2.29M (24%) identified by BS-SNPer as non-CpG; this is largely expected, given that at a CG-to-TG mutation half of the reads are uninformative for genotyping under bisulfite conditions. Accordingly, even after adding and removing CpGs called by BS-SNPer, our calculated global methylation for the CAST metasample (68.2%) remained markedly lower than those obtained from personal alignments (72–73%), indicating quantification bias was incompletely addressed. Together, our results suggest that bisulfite genotyping was insufficient for addressing CpG variation between our divergent strains, at our sequencing depth.

**Table 4.** BS-SNP results on pooled CAST samples

BL6-unique CpGs	2 285 477
BL6-unique CpGs identified by BS-SNPer	542 642
CAST-unique CpGs	2 266 009
CAST-unique CpGs identified by BS-SNPer	1 153 611

Another potential approach to dealing with CpG variation is to align to the standard reference, then filter out known variation based on an external database, such as dbSNP. For example, one could exclude any CpG overlapping known variants of certain criteria (high-population frequency, CG-to-TG, etc.). Assuming a sample has a relatively small number of private SNPs, this method would theoretically remove the main source of hypomethylation bias and indeed this method has been previously used in literature, albeit on much less distant genotypes (31). However, stringent filtering could overestimate the amount of variation and remove a proportion of CpGs that remain in the sample, in addition to truly lost CpGs. Furthermore, non-CpG differences from the reference would not be addressed by this method.

**Figure 5.**
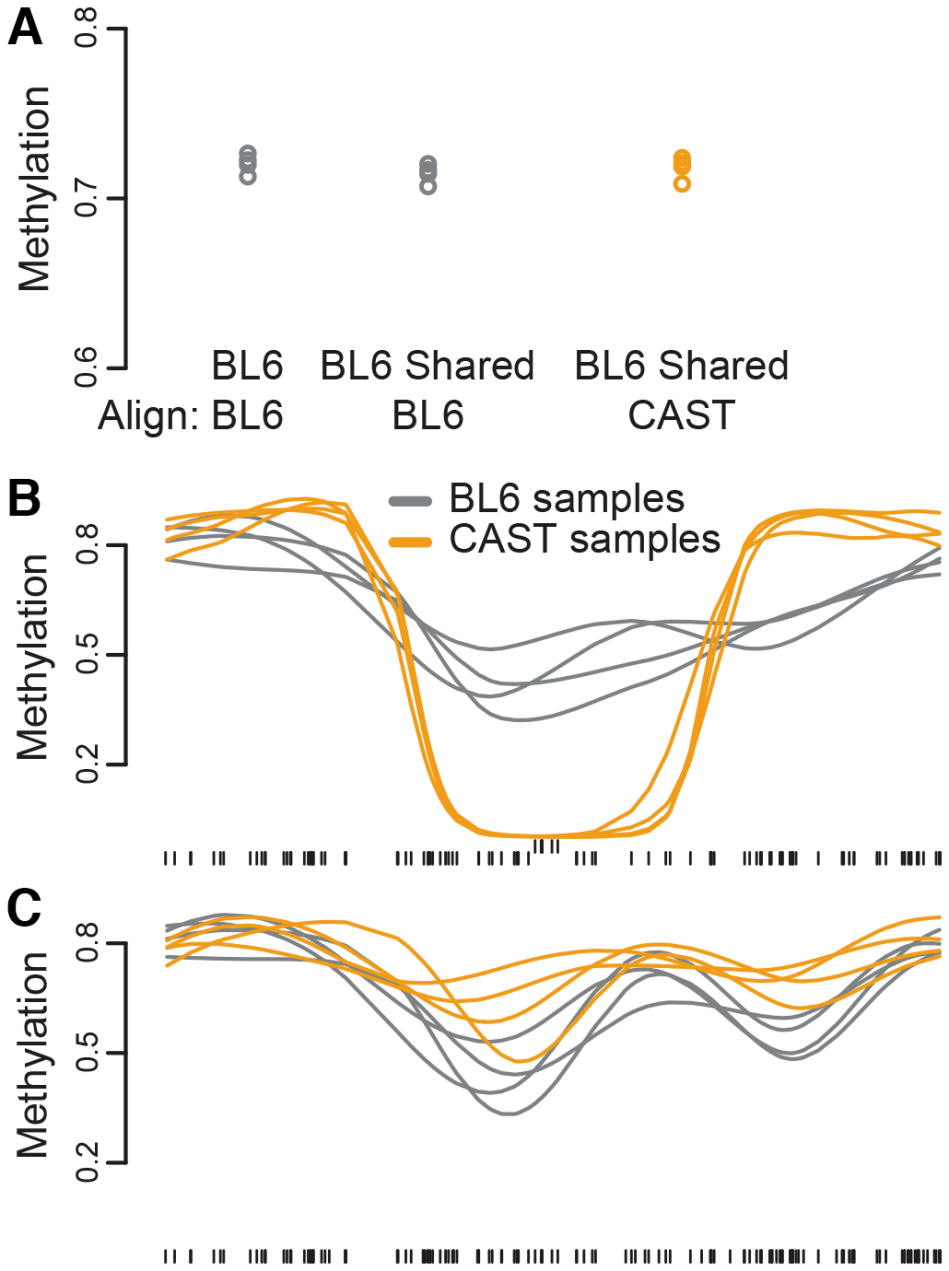
Variation filtering mostly, but not completely, removes quantification bias. Filtering out strain-unique CpGs from the BL6 reference sufficiently addresses global methylation bias (**A**). However, an example 11 kb genomic region, despite containing no CpGs unique to either mouse strains, appears as a DMR under the filtering strategy (**B**) despite no true methylation difference under personal-genome alignment (**C**). This is due to misalignment of reads: upwards tick marks indicate CpGs with average coverage >100 in the filtered analysis.

To test this ‘variation filtering’ approach, we aligned CAST samples to the BL6 reference genome, then removed all BL6-unique CpGs from the dataset prior to smoothing; this represents a ‘perfect’ adjustment, in which all CpG variation is removed with no overestimation. As expected, this method removed the bias in global methylation, with estimates close to those obtained with personal genomes (Figure 5A). Focal analysis identified 622 DMRs (Supplementary Table S7); of these, 529 overlapped with the 716 DMRs identified in the personal, unique-removed analysis, indicating that differential finding under this method is at least somewhat comparable, though with some loss of power. However, care must still be taken when examining results obtained with this approach, as non-CpG-related alignment errors can still produce false-positive regions. As an example, we found a region on chr10 containing no strain-unique CpGs which appeared to be greatly hypomethylated in CAST samples when aligned to the BL6 reference, but showed no methylation difference when samples were aligned to personal references (Figure 5B and C). We discovered that this result was due to thousands of unmethylated CAST mitochondrial reads, which aligned preferentially to the BL6 chr10 rather than to the BL6 chrM due to SNPs in both regions, but aligned correctly to chrM when the CAST reference was used (Figure 5C). Overall, these results indicate that while variation filtering is sufficient to address large-scale quantification bias, residual alignment errors may still create local false-positive regions.

### Addressing heterozygosity

Thus far, we have discussed the implications of CpG variation within the relatively narrow context of a homozygous genome—namely, methylation experiments involving inbred genotypes, most relevant to studies involving animal and plant species such as mouse or *Arabidopsis*. In most other scenarios, including all human studies, genomes are generally heterozygous. Accurate quantification of methylation at heterozygous CpGs is an ongoing and challenging problem: reads from a C-to-T allele must be distinguished from truly unmethylated reads from the CpG-containing allele. This requires reads containing both the CpG and a phased variant, which is unlikely with current short read sequencing technologies and the reduced complexity of bisulfite space; as well as a method of aligning to a diploid reference genome, which standard aligners do not support.

However, even in the presence of heterozygosity, applying knowledge of CpG variation and personalized genomes remains useful. We propose the following algorithm for WGBS analysis of heterozygous samples:

- Create personalized genomes for each sample; for heterozygous loci, choose one allele to include in the genome.
- Align samples to their personalized genomes and place CpGs into a common coordinate space.
- For each individual sample, remove any CpG site which is heterozygous in that sample.
- Smooth methylation values over the remaining CpGs, including any CpGs that are homozygous sample-unique.

Our analysis of data from inbred mice shows that this approach removes all bias and is almost as powerful as having phased reads. Specifically, step 1 creates a personalized genome with improved accuracy at homozygous variants relative to the standard reference. Accuracy over heterozygous loci remains partial; however, as we observed previously in our analysis of variation filtering, methylation bias resulting purely from alignment errors is both rare and highly localized (Figure 5). That analysis also showed that removal of CpG sites susceptible to quantification errors is far more important in addressing bias; such sites are removed in step 3. The result is a method that preserves information at homozygous sample-unique CpGs, while removing locations contributing to quantification bias.

### An application to human cancer

One potential non-strain application of our heterozygosity method is in normal-cancer comparisons; as cancer samples are known to contain a multitude of genomic mutations, they represent samples especially likely to be substantially distant from the standard human reference genome. As an example of this scenario, we obtained publicly available WGBS data of two samples, normal human brain and a human glioblastoma cell line U87MG, from a study on methylation of super-enhancers (25); as well as publicly available SNP and indel information on the U87MG line, relative to the human reference genome, from a separate study (26). The U87MG genome was annotated as containing 2 384 078 SNPs and 320 051 indels; 1 116 438 SNPs and 172 183 indels were marked as heterozygous. These resulted in 334 625 CpGs being lost relative to the human reference, while adding 331 108 U87MG-unique CpGs. This number of affected CpGs is about 10-fold lower than we observed in the BL6-CAST comparison, but nevertheless constitutes around 1% of CpG sites in the human genome.

Using our pipeline for personalized alignment and estimation in the presence of heterozygosity, we mapped U87MG reads to a personalized U87MG genome created from the above variant information. Both the U87MG and normal brain samples were also aligned to hg19. All alignments were placed into the hg19 coordinate system; then, prior to smoothing, 148 185 heterozygous CpGs were filtered from the U87MG-aligned U87MG sample. Due to lack of replicates, we could not perform standard DMR finding; instead, we measured methylation across tiled genomic windows.

Unlike our observations in CAST mice, U87MG global methylation was not strongly affected by reference genome choice, with only about a 0.3% difference between alignments. Quantification bias was also not readily apparent at larger scales; of 255 236 covered 10-kb windows tiled across the genome, only 66 contained a mean methylation disparity between alignments >10%. These results were expected, given that the U87MG genome had a relatively smaller number of ‘lost’ CpGs compared to the CAST genome; furthermore, as the cancer sample had a low starting methylation, the effect of 0%-methylated sites on the mean would naturally be smaller. However, methylation bias was still clearly present on the local scale: when the genome was tiled at 1-kb rather than 10-kb, 6647 of the 2 409 180 resulting windows now showed a methylation disparity above the 10% threshold. The majority of these windows (5361 or 81%) showed lower U87MG sample methylation in the hg19 alignment relative to the U87MG alignment, as expected from the effect of ‘lost’ CpGs on estimation.

**Figure 6.**
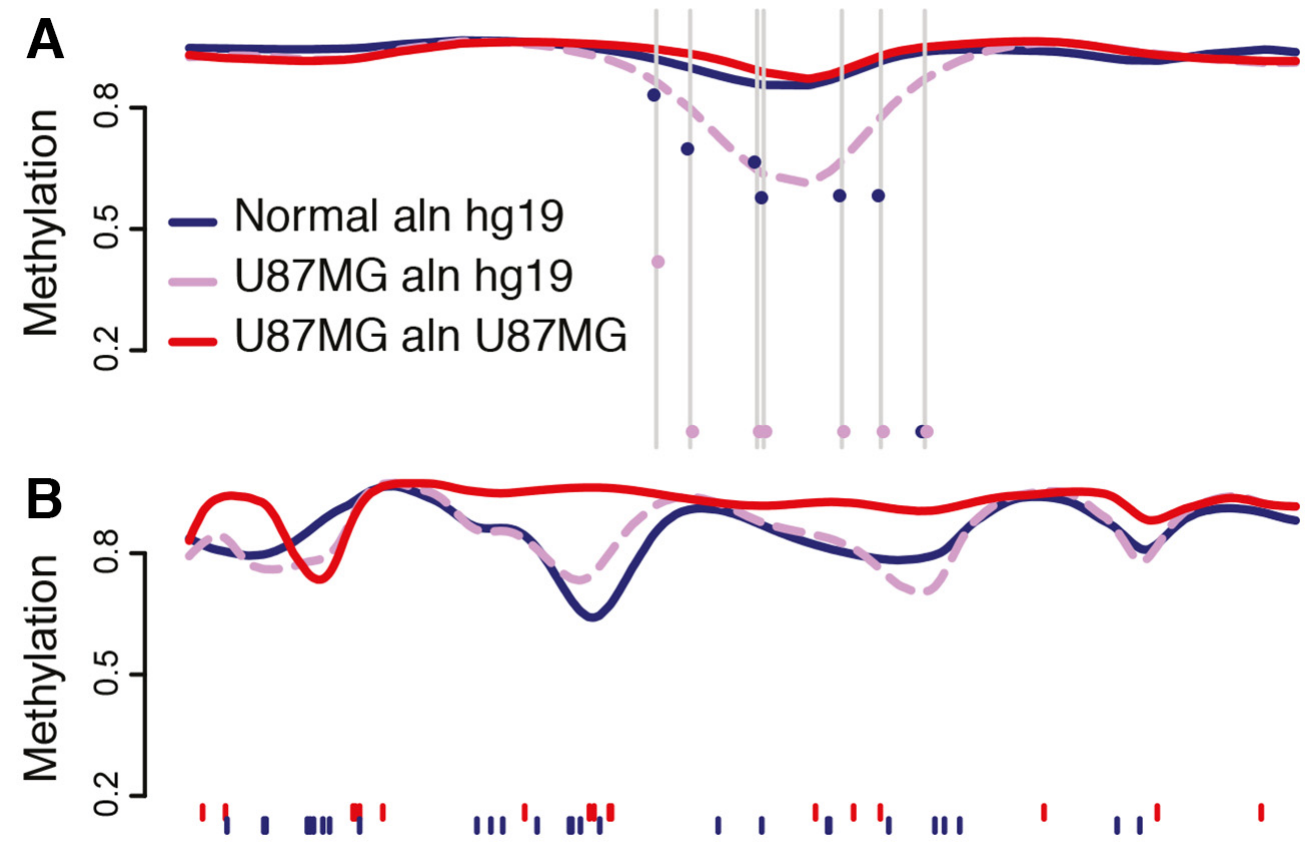
Impact of self-alignment in a normal-cancer comparison. (**A**) A 5 kb window across DHX37 displays false-positive hypomethylation that is ameliorated with U87MG self-alignment. Grey lines mark CpGs unique to hg19; dots indicate raw methylation levels in U87MG and hg19 alignments. (**B**) A 25 kb window across the LGALS8 gene shows markedly higher smoothed methylation in U87MG when self-aligned. Tick marks indicate CpGs unique to hg19 (blue) or U87MG (red).

As we saw with our inbred mouse data, aligning the U87MG sample to its own reference appeared to eliminate some false-positive methylation differences. Of 1 725 532 1-kb windows that displayed a >10% methylation difference between normal and U87MG samples on the hg19 alignment, 27 864 (1.6%) no longer showed the same large methylation difference when the normal sample was compared to the U87MG-aligned U87MG sample. As an example, a roughly 1-kb region across the gene body of DHX37 appeared to be hypomethylated in the U87MG sample relative to normal brain when the sample was aligned to hg19. However, this was caused by 0%-methylation calls at six CpG positions in the region that were lost in the U87MG sample; correspondingly, when U87MG was self-aligned, there was virtually no difference between samples (Figure 6A). As DHX37 has been previously linked to neurologic disease (32,33), we found this an especially compelling case of alignment bias producing an appealing but false-positive candidate gene.

Conversely, and again as observed in our mouse data, self-alignment could result in novel identification of regions of interest. The 16 699 tiled 1-kb windows indicated a normal-cancer methylation difference >10% only when the U87MG sample was self-aligned. As an example, a 25-kb region on chr1 containing a number of both hg19-unique and U87MG-unique CpGs contained several regions of hypermethylation in U87MG when the sample was self-aligned (Figure 6B). This region covers part of the LGALS8 gene, a galectin previously implicated in migration and proliferation of U87 glioblastoma cells (34). Thus, it is plausible that self-alignment would result in finding more normal-cancer DMRs in relevant genes, increasing the power of the study. Overall, our results suggested that self-alignment of the U87MG cancer sample could correct local alignment bias and facilitate more comprehensive identification of regions of interest, even if not at the scale seen in our extremely distant mouse strains.

## DISCUSSION

Here we have studied the unique challenge of comparing DNA methylation data between divergent genotypes. We have introduced a method which, through imputation, allows the inclusion of genotype-specific CpGs in regional methylation analysis. The most powerful version of our method uses personalized genomes, accounting for differences in genomic coordinates, and we show that this results in increased power for detection of DMRs while dramatically reducing bias. We expect our method to have the most significant implications for studies using model organisms, which often have widespread and consistent differences between genotypes, but it is widely applicable to any setting where genetic variation is expected between sample groups.

We have compared our method to various other potential strategies for handling data from distant genotypes. At the most extreme end, using just a single reference genome results in a massive quantification bias that completely obscures the true signal. We have for the first time precisely quantified this bias by also aligning to the distant sample’s reference genome, observing a near-total inversion of bias both globally and locally. As a first step to detect bias of this nature, we recommend routinely examining global methylation across samples. This is easy and effective, particularly as the proportion of genotype-specific CpGs in a sample increases. Note that bias is not always the only explanation for global differences; for example, it is well-established that global methylation is cell type dependent.

Naturally, personalized genomes are not always available, especially outside the context of model organisms; however, in these cases, any measure to account for variation will still improve analysis. If a database of sequence variants is available, methylation calls can be filtered post-alignment to remove CpGs not found in all genotypes. This can be done individually for each sample at little computational cost. Alternatively, existing tools such as Bis-SNP or BS-SNPer can genotype samples using bisulfite-converted DNA. As we observed, the success of such tools depends on both the coverage of the experiment and the extent of CpG variation; while our attempts in inbred mouse strains largely failed at 28× coverage, the tools have been shown to successfully capture smaller-scale variation in human data.

When partial adjustments (or, inadvisably, no adjustments) are made for CpG variation, identified regions of interest should be routinely scrutinized to rule out the possibility of quantification bias or alignment errors. C-to-T mutations are often easy to spot, appearing as completely unmethylated sites (i.e. exactly 0% rather than near-0%) within otherwise highly methylated regions. Additionally, as we observed, sites of unusually high coverage can be indicators of misalignment. Regions with one or both of these characteristics should be carefully validated to ensure they are not false positives.

The exact effect of quantification bias depends critically on experimental design. Here, we have focused on an analysis where the goal is to compare two distinct genotypes. In contrast, a study might distribute genotypes evenly among groups of comparison, e.g. drug versus placebo; this design is common in studies involving human subjects. In these experiments, rather than skew methylation levels in a specific group, quantification bias would increase within-group variation, consequently causing a loss of power. The strategy we advocate of personalized alignment addresses both situations, both removing bias and decreasing variation. In general, most studies in model organisms involving multiple strains will require the availability of personalized genomes. Such studies are often undertaken specifically to understand the impact of genotype, thus the accurate comparison of groups with vastly different genomes is paramount. This is for example the rationale behind the development of the mouse Collaborative Cross (35). *Arabidopsis thaliana* is frequently used for the same purpose; although plants have extensive non-CpG methylation, we expect variation across those sites to be governed by similar principles.

When moving beyond homozygous-inbred organisms, allele heterozygosity presents a few additional considerations. Accurate alignment requires a diploid reference genome, and accurate quantification at heterozygous CpGs further requires that non-CpG reads be distinguishable from unmethylated reads. In principle, the eventual solution to these issues involve aligning to both haplotypes and analyzing each haplotype separately. This would require advances in alignment tools as well as phased genomes, such as those made possible by 10× Genomics. In the interim, however, we have developed and presented our method to correct for known heterozygous mutations across CpGs—by far the largest source of quantification bias—namely by filtering such CpGs from analysis.

We have applied our approach to heterozygous CpG variation to a human disease context, using existing bisulfite data and variant information from normal brain and a glioblastoma cell line. Although on a smaller scale of magnitude than our highly divergent mouse strains, we show that simply aligning the cancer line to its personalized genome rather than the human reference profoundly affects methylation quantification. Importantly, this is enough for us to identify both false-positive regions driven by bias and novel regions previously obscured by bias, even in the presence of heterozygosity. Although our cancer application is selected from existing data and thus lacks replicates or variant data for the normal sample, these are easily obtained in a full cancer experiment, i.e. through increased sample size and genotyping of samples.

In human studies, any two individuals will be genetically closer than the two mouse strains studied here, and the rate of CpG variation less extreme. However, we know from genome-wide association studies that individuals in a group are rarely random samples from a background distribution. Hence, quantitation bias may still exist and require correction, e.g. if certain CpGs are consistently lost in one group relative to the other. Correction becomes particularly crucial when widespread genetic differences between sample groups are not only likely but functionally relevant—most notably, in cancer-normal studies such as the one we examined.

## CONCLUSION

We have shown that quantification bias stemming from CpG variation between samples can severely affect analysis of bisulfite converted DNA. We have proposed a method which addresses this, and furthermore integrates genotype-specific CpGs into differential methylation analysis to increase power. Our method requires either personalized reference genomes, or detailed knowledge of sequence variation across CpGs. Future studies employing bisulfite sequencing should carefully consider this issue, especially if the goal is comparison between distinct genotypes.

## DATA AVAILABILITY

WGBS data is available under NCBI GEO accession number GSE87101. This includes alignments of the samples to both CAST and BL6 genomes, expressed in BL6 coordinates as well as alignment of the samples to the CAST genome, expressed in CAST coordinates. Supplementary Methods contain details on the experimental protocol.

## Supplementary Material

gkz674_Supplemental_Files
