# Supplementary material for: Analyzing whole genome bisulfite sequencing data from highly divergent genotypes
Source: Nucleic Acids Res. 2019 Aug 8;47(19):e117. doi: 10.1093/nar/gkz674 (PMC6821270; doi:10.1093/nar/gkz674)
Supplement: gkz674_Supplemental_Files [file gkz674_supplemental_files.zip › Wulfridge_SupplementaryMaterials.pdf]

Supplementary Materials

for

Analyzing whole genome bisulfite sequencing data from highly divergent genotypes

Phillip Wulfridge, Ben Langmead, Andrew P. Feinberg, Kasper D. Hansen*

Contents

1. Supplementary Methods.
2. Supplementary Discussion.
3. Supplementary Figures S1-S5.
4. Supplementary Tables S3-S5.

*To whom correspondence should be addressed. Email: khansen@jhsph.edu

Supplementary Methods

Sample information

Liver samples from two mouse strains (C57BL/6J and CAST/EiJ, 4 mice per strain) were obtained from Jackson Laboratories. All mice were 6-week-old females.

Genomic mutation rate

The MOD file for CAST from UNC [1] lists 20,539,633 bp of single nucleotide variants, 6,633,124 bp of insertions, and 5,279,608 bp of deletions relative to BL6 (including autosomes, allosomes, and chrM). The length of the BL6 genome is 2,654,911,517 bp, for an overall genomic mutation rate of (20,539,633 + 6,633,124 + 5,279,608) / 2,654,911,517 = 1.2%.

DNA extraction and sequencing

Genomic DNA was extracted from liver using the Qiagen DNEasy kit, with an additional RNase incubation step (50 μg/sample, 30 minutes) prior to column application to remove RNA.

WGBS single indexed libraries were generated using the TruSeq DNA LT Sample Preparation Kit (Illumina) according to the manufacturer's instructions with modifications. 1μg gDNA for BL6 samples (1.34μg gDNA for CAST samples due to observed partial DNA degradation) was quantified via Qubit dsDNA BR assay (Invitrogen) and 0.8% agarose gel. 1% Unmethylated lambda DNA (cat#D1521, Promega) was spiked in for monitoring bisulfite conversion efficiency. Samples were fragmented by Covaris S2 sonicator to an average insert size of 350bp (80sec, Duty cycle 10%, Intensity 5, Cycles per burst 200). Size selection was performed using AMPure XP beads and insert sizes of 300-400bp were isolated. Samples were bisulfite converted after size selection using EZ DNA Methylation-Gold Kit (cat#D5005, Zymo) following the manufacturer's instructions. Amplification was performed following bisulfite conversion using Kapa Hifi Uracil+ (cat#KK282, Kapa Biosystems) polymerase and cycling conditions: 98degC 45s /8cycles: 98degC 15s, 65degC 30s, 72degC 30s / 72degC 1 min.

Final libraries were confirmed via 2100 Bioanalyzer (Agilent) High-Sensitivity DNA assay. Libraries were quantified by qPCR using the Library Quantification Kit for Illumina sequencing platforms (cat#KK4824, Kapa Biosystems), using 7900HT Real Time PCR System (Applied Biosystems). Libraries were sequenced on an Illumina HiSeq2000 sequencer using 100bp paired-end runs with a control lane.

Pyrosequencing validation

For validation, we performed pyrosequencing of differentially methylated regions from the mouse analysis. We selected 3 such regions, within the Aldh16a1, Tdgf1, and Eif2ak3 genes. As described in the Results, all 3 regions were selected as part of the "unique-included" list of DMRs, and 1 (Eif2ak) was selected to not be part of the "unique-removed" list of DMRs. Within these regions, CpGs were selected for validation based on accessibility for pyrosequencing, using the PyroMark Assay Design from Qiagen. The list of PCR forward and reverse primers and sequencing primers for each region is provided in Supplementary Table S5. First, DNA extraction of liver genomic DNA was reperformed using Quick-DNA/RNA Miniprep Plus kit (cat#D7003, Zymo). Bisulfite conversion of DNA samples was performed via EZ DNA Methylation-Lightning kit (cat#D5030, Zymo). Following this, regions were amplified using the Qiagen PyroMark PCR kit and pyrosequencing performed on a Qiagen PyroMark Q48 machine. Methylation was quantified at each site using Qiagen Pyromark Q48 Autoprep software version 2.4.2 in CpG analysis mode.

Overlap with functional regions

Genomic intervals for ENCODE/LICR histone [2] and TFBS tracks [3], RefSeq genes [4], and CpG islands [5] were obtained via the UCSC Genome Browser. Histone and TFBS data were generated by the ENCODE Consortium [6] and the Bing Ren laboratory, and are also available at GEO accessions GSE31039 and GSE36027. ENCODE filenames as listed in the UCSC download server are provided in Supplementary Table S4. Promoter regions of Refseq genes were defined as the 5-kb region flanking a gene's transcription start site. CpG shores were defined as the 2-kb regions upstream and downstream of a CpG island.

Given a set of DMRs as well as a class of regions, we computed the odds ratio of enrichment by considering the overlap in CpGs between the two sets of regions, accounting for the fact that not all CpGs were measured in our data. This approach naturally addresses issues of non-uniform distribution of CpGs.

Supplementary Discussion

Pyrosequencing

Pyrosequencing results are presented in Supplementary Figures S3-S5. Supplementary Figures S3 and S4 (around the *Aldh16a1* and *Tdgf1* genes) are two regions differentially

methylated regions which are found using both our "unique-included" and "unique-removed" approaches, whereas Supplementary Figure S5 (around the *Eif2ak3* gene) is an example of a DMR only found using our "unique-included" approach. Broadly, the pyrosequencing validates our WGBS analysis results. The various regions all exhibit differential methylation in the same direction in both assays. Supplementary Figure S4 (around the *Tdgf1* gene) needs further comment: there appears to be a difference in magnitude of the methylation levels between the smoothed WGBS curves and the pyrosequencing results, but only for the BL6 samples. In our experience and opinion, we expect some difference between the two assays, but contrasts between samples groups are often highly concordant.

We hypothesized that this might be related to smoothing. In panels (a) and (c) of Supplementary Figures S3-S5, we depict the unsmoothed WGBS data. We stress the WGBS data has low overall coverage (7-8x per sample) and a coverage in the Tdgf1 region of 3.8-6.7x with a coverage of the individual CpGs we use for pyro at 1.6-13x. This implies we have very high technical variance when considering a single, unsmoothed CpG. The plots of the unsmoothed data reveal that the smoothed WGBS data faithfully recreates the broad pattern of methylation, with some variation along the smoothed line. Furthermore, when reviewing the 6 individual CpGs assayed by pyro, we see much the same pattern, especially when low coverage (samples, CpGs) are excluded. We conclude that the two assays agree.

Bibliography

[1] *UNC Systems Genetics*. http://csbio.unc.edu/CCstatus/index.py?run=Pseudo.

[2] *ENCODE/LICR Histones*. https://genome.ucsc.edu/cgi-bin/hgTrackUi?g=wgEncodeLicrHistone. (Visited on 09/13/2016).

[3] *ENCODE/LICR TFBS*. https://genome.ucsc.edu/cgi-bin/hgTrackUi?g=wgEncodeLicrTfbs. (Visited on 09/13/2016).

[4] *Refseq genes*. https://genome.ucsc.edu/cgi-bin/hgTrackUi?g=refGene. (Visited on 07/14/2016).

[5] *CpG Islands*. https://genome.ucsc.edu/cgi-bin/hgTrackUi?g=cpgIslandSuper. (Visited on 07/15/2016).

[6] ENCODE Project Consortium. An integrated encyclopedia of DNA elements in the human genome. *Nature* 489 (2012), 57–74. DOI: 10.1038/nature11247.

Supplementary Figures

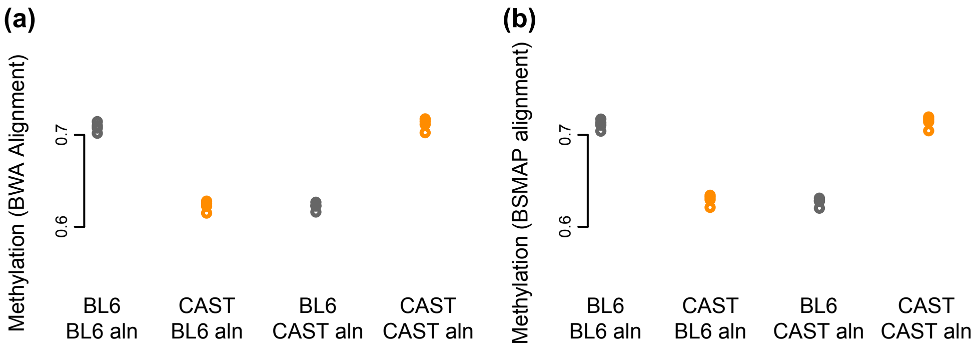

Supplementary Figure S1. Global methylation bias in other alignment methods. Global methylation estimates of BL6 and CAST samples aligned to BL6 or CAST reference genomes with either BWA **(left)** or BSMAP **(right)** aligners. Hypomethylation bias is observed in samples aligned to a distant reference genome regardless of the aligner used.

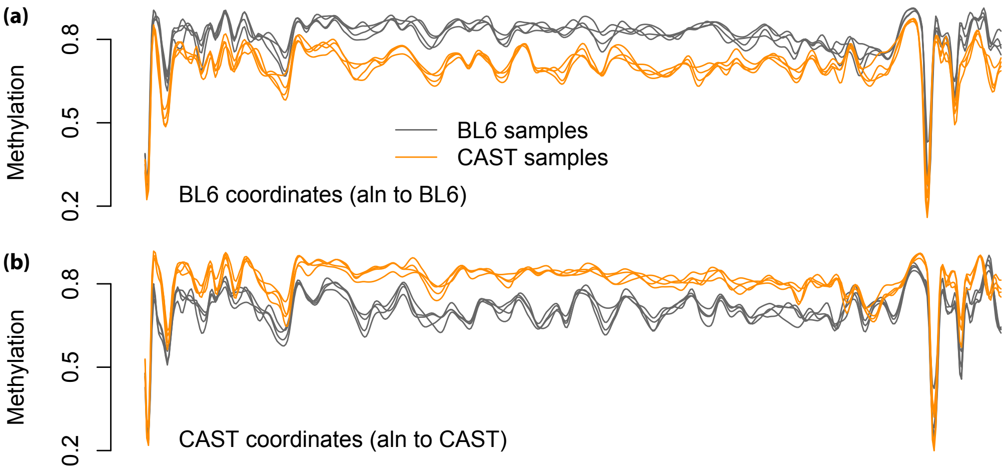

Supplementary Figure S2. Mapping bias causes false-positive large-scale methylation changes. The same 2.4 Mb genomic region is depicted in two different coordinate systems and with different data processing. **(a)** All samples aligned to BL6. **(b)** All samples aligned to CAST.

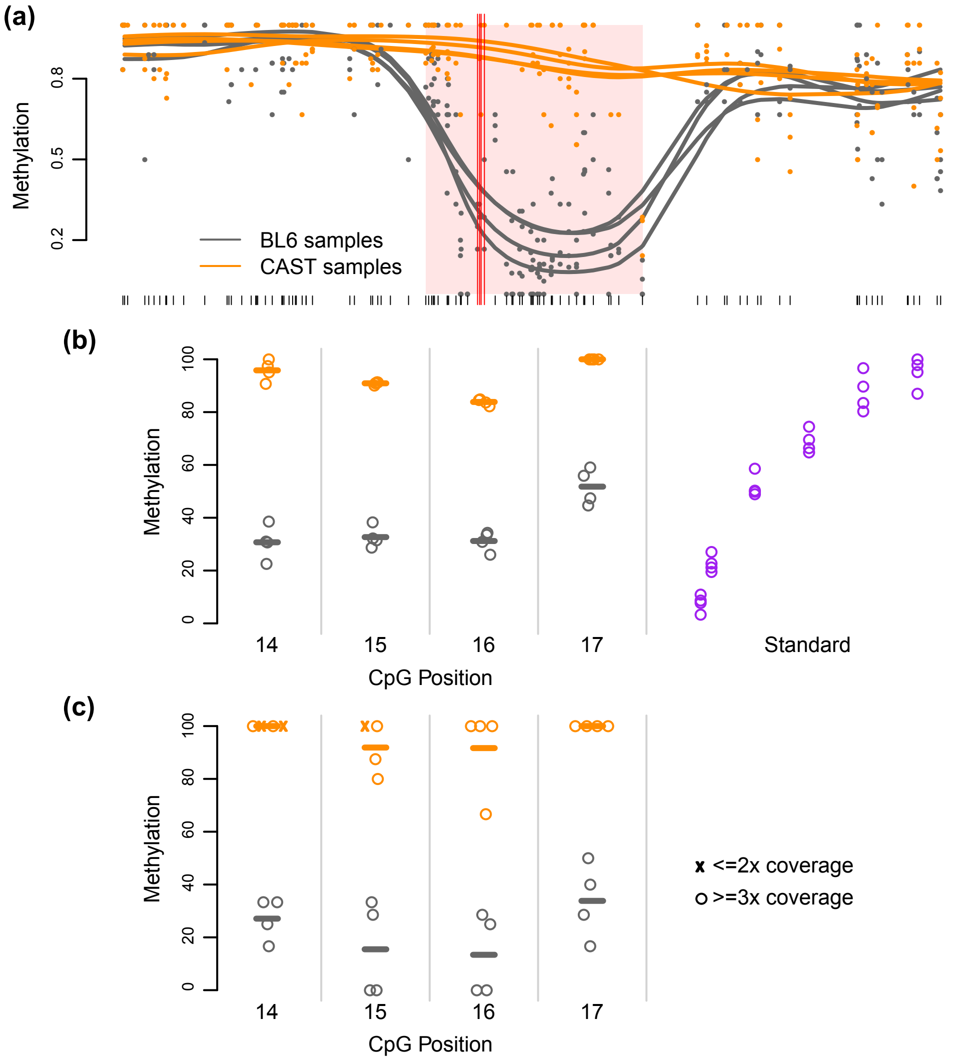

Supplementary Figure S3. Pyrosequencing validation of a strain DMR around the *Aldh16a1* gene. (a) Smoothed and raw WGBS data (DMR highlighted in pink). Each line represents the smoothed methylation curve of a sample, using the "unique-included" approach. Individual colored points represent a raw methylation measurement at a CpG; recall that the WGBS data has a coverage of 7-8x. 4 CpGs assayed by pyrosequencing are highlighted with red lines. **(b)** Pyrosequencing for 4 selected CpGs, including standardization controls at 0%, 5%, 25%, 50%, 75%, and 100%. **(c)** Raw methylation values as estimated from WGBS data across the same 4 selected CpGs. Horizontal bars indicate means for each sample.

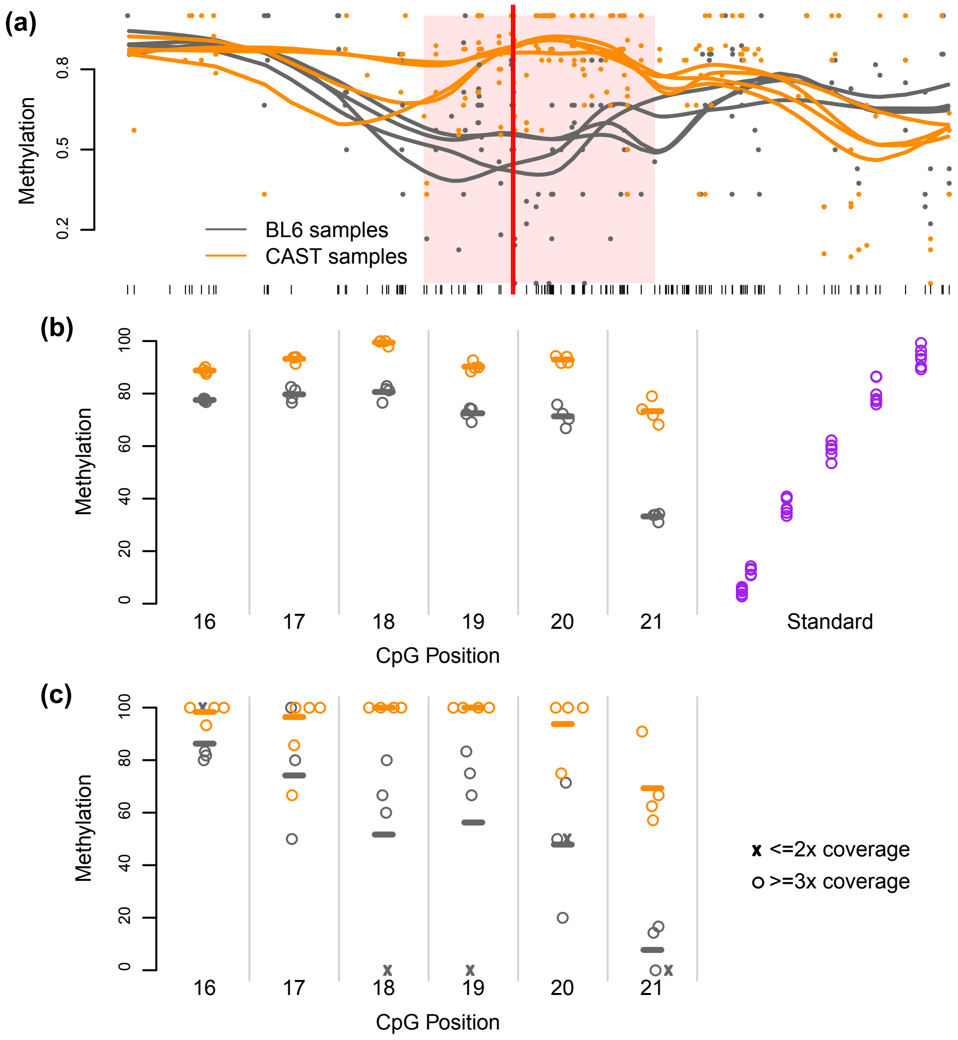

Supplementary Figure S4. Pyrosequencing validation of a strain DMR around the *Tdgf1* gene. (a) Smoothed and raw WGBS data (DMR highlighted in pink). Each line represents the smoothed methylation curve of a sample, using the "unique-included" approach. Individual colored points represent a raw methylation measurement at a CpG; recall that the WGBS data has a coverage of 7-8x. 6 CpGs assayed by pyrosequencing are highlighted with red lines. **(b)** Pyrosequencing for 6 selected CpGs, including standardization controls at 0%, 5%, 25%, 50%, 75%, and 100%. **(c)** Raw methylation values as estimated from WGBS data across the same 6 selected CpGs. Horizontal bars indicate means for each sample.

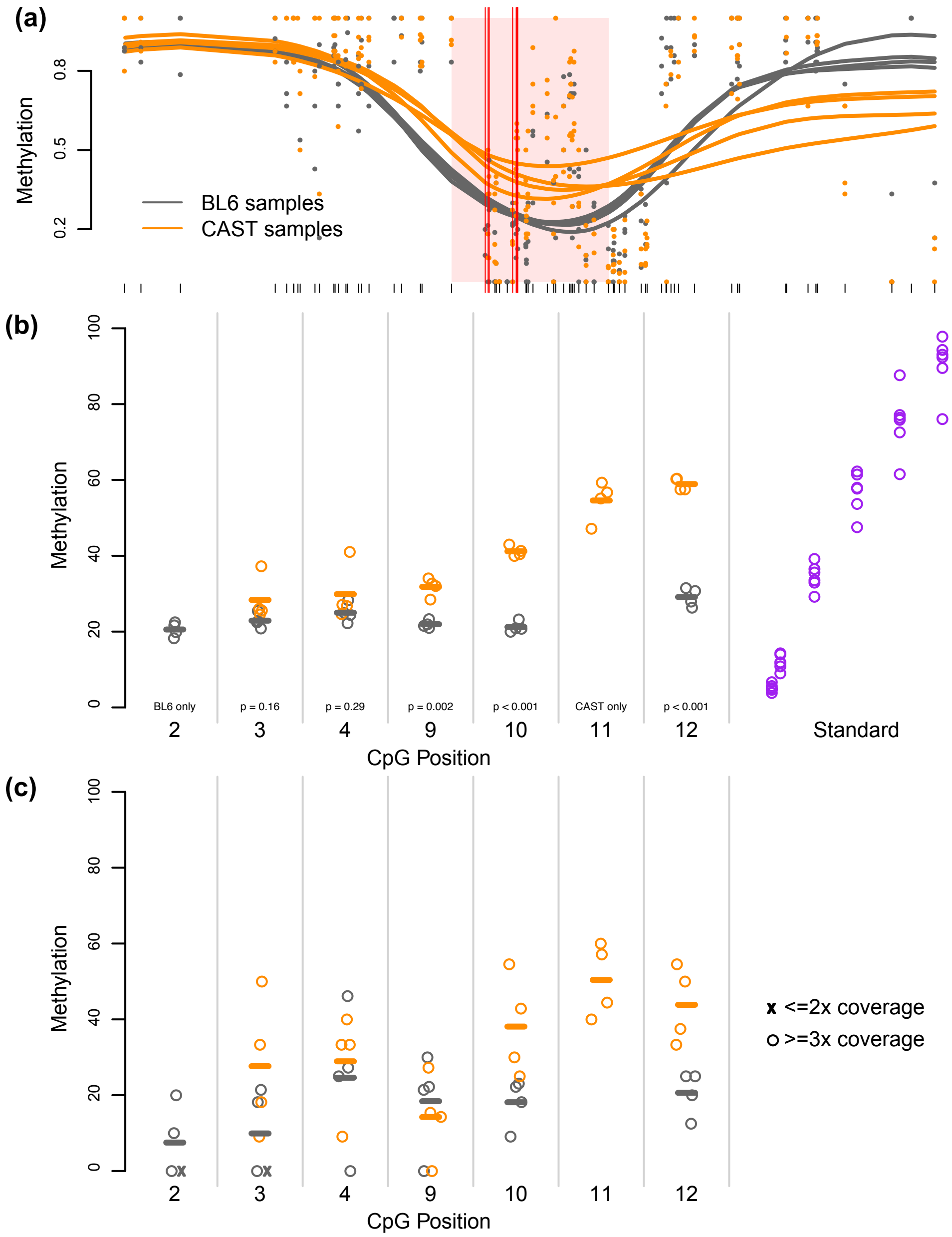

Supplementary Figure S5. Pyrosequencing validation of a strain DMR around the *Eif2ak3* gene. This DMR was identified exclusively by the unique-included method. **(a)** Smoothed and raw WGBS data (DMR highlighted in pink). Each line represents the smoothed methylation curve of a sample, using the "unique-included" approach. Individual colored points represent a raw methylation measurement at a CpG; recall that the WGBS data has a coverage of 7-8x. 7 CpGs assayed by pyrosequencing are highlighted with red lines. **(b)** Pyrosequencing for 7 selected CpGs, including standardization controls at 0%, 5%, 25%, 50%, 75%, and 100%. **(c)** Raw methylation values as estimated from WGBS data across the same 7 selected CpGs. Horizontal bars indicate means for each sample. Note that 2 of the 7 CpGs are only present in one of the two strains.

Supplementary Tables

Supplementary Table S3. Enrichment of strain-specific DMRs in genomic features.

Feature	$\log_2$(OR) Shared	$\log_2$(OR) Included only	$\log_2$(OR) Removed only	p-value Shared	p-value Included only	p-value Removed only
Refseq promoters	0.98	0.55	0.23	$< 2.2 \cdot 10^{-16}$	$< 2.2 \cdot 10^{-16}$	0.046
CpG Islands	0.52	-0.42	0.099	$< 2.2 \cdot 10^{-16}$	$4.76 \cdot 10^{-5}$	0.59
CpG Shores	1.21	1.09	-1.52	$< 2.2 \cdot 10^{-16}$	$< 2.2 \cdot 10^{-16}$	$3.4 \cdot 10^{-10}$
H3K4me1	3.11	2.92	3.81	$< 2.2 \cdot 10^{-16}$	$< 2.2 \cdot 10^{-16}$	$< 2.2 \cdot 10^{-16}$
H3K4me3	1.99	1.48	2.16	$< 2.2 \cdot 10^{-16}$	$< 2.2 \cdot 10^{-16}$	$< 2.2 \cdot 10^{-16}$
H3K27ac	1.97	1.96	2.60	$< 2.2 \cdot 10^{-16}$	$< 2.2 \cdot 10^{-16}$	$< 2.2 \cdot 10^{-16}$
CTCF	1.59	1.74	2.14	$< 2.2 \cdot 10^{-16}$	$< 2.2 \cdot 10^{-16}$	$< 2.2 \cdot 10^{-16}$
Pol2	0.32	-1.28	1.33	$1.2 \cdot 10^{-7}$	$< 2.2 \cdot 10^{-16}$	$1.5 \cdot 10^{-15}$
Any feature above	3.09	2.87	3.54	$< 2.2 \cdot 10^{-16}$	$< 2.2 \cdot 10^{-16}$	$< 2.2 \cdot 10^{-16}$

Supplementary Table S4. Filenames for ENCODE data.

Filename
`wgEncodeLicrHistoneLiverH3k27acMAdult8wksC57bl6StdPk.broadPeak`
`wgEncodeLicrHistoneLiverH3k4me1MAdult8wksC57bl6StdPk.broadPeak`
`wgEncodeLicrHistoneLiverH3k4me3MAdult8wksC57bl6StdPk.broadPeak`
`wgEncodeLicrTfbsLiverCtcfMAdult8wksC57bl6StdPk.broadPeak`
`wgEncodeLicrTfbsLiverPol2MAdult8wksC57bl6StdPk.broadPeak`

Supplementary Table S5. Primers for pyrosequencing.

Gene	Primer set	Primer	Sequence
Eif2ak3	1	Forward	TTTTGTGGTGTGAGGAATTAGAT
		Reverse	ACCCACATACTCAACTTTAACTTACTATA
		Sequencing	ACTACTAACACTTAACCAAAT
Eif2ak3	2	Forward	AGTTAGAAAGATTGTAGTGGGAATAAGA
		Reverse	ACTACACAAAAAAACCCTATCTCT
		Sequencing	ATATTATTAGAGAAAAATAAATT
Aldh16a1	1	Forward	TTTTAGGTTGGGAGGTTAGGATAGG
		Reverse	ACTAACACCTAAACCCCTAAATATACA
		Sequencing	AGTTGAGTTAGGAGGA
Tdgf1	1	Forward	TTGTATGGGTTGGAGAAGATT
		Reverse	ATCTTACAACCTAATCAAAACTACCT
		Sequencing	GGGTTGGAGAAGATTT

Additional table descriptions

Supplementary Tables S1, S2, S6, and S7 contain lists of differentially methylated regions (DMRs). Specifically,

- Supplementary Table S1 contains DMRs for the biased analysis (all samples aligned to BL6, no adjustment for CpG variation);
- Supplementary Table S2 contains DMRs for the unique-included analysis (samples aligned to personal genomes, sites of CpG variation retained);
- Supplementary Table S6 contains DMRs for the unique-removed analysis (samples aligned to personal genomes, sites of CpG variation removed);
- Supplementary Table S7 contains DMRs for the variation filtering analysis (all samples aligned to BL6, sites of CpG variation removed).

These files are all expressed in BL6 coordinates, in genome version `mm9`. The coordinates are 1-based. The columns are as follows

- `Chromosome, Start, End`: chromosomal coordinates.
- `# CpGs, Width (bp)`: The number of CpGs in the region and the width of the region in base pairs (as measured on the mm9 coordinate system).
- `Area T-Stat`: The sum of the t-statistics across all CpGs within the region. This is used for family-wise error rate measurements, where it is compared to the area T-stats of null permutation DMRs.
- `BL6 Mean, CAST Mean`: The average methylation levels in BL6 and CAST.
- `Mean Difference`: The calculated mean methylation difference between BL6 and CAST (on the beta scale) across the region. A positive number indicates higher methylation in CAST.
